# Variations of Using Teeth in Various Animals

**Published:** 1889-01

**Authors:** F. S. Buckley

**Affiliations:** U. of M.


### "What Variations in the Manner of Using the Teeth Are Exhibited by Various Animals Including Man."

BY F. S. BUCKLEY, U. OF M.

The subject comprises the entire animal kingdom, vertebrate and invertebrate.

All vertebrate animals have teeth except birds and the lowest order of fishes. Moreover, these are all calcified teeth except one order of fishes which has horny teeth. Birds also use horny bills for the same purpose as teeth, and several genera of extinct birds have true calcified teeth.

The invertebrate animals have horny or chitinous teeth except the three lowest orders which use the jaws for the same purpose.

The various uses of the teeth are divided by Dr. Owen into primary and secondary. Primary uses—seizing, dividing, lacerating, and triturating the food. Secondary uses—subserviency to speech, as ornaments or as characterizing age or sex, as inflictors of wounds in combat and in defense, aids to locomotion, means of anchorage and implements for transporting and working of building materials.

With some possible exceptions the primary uses are present in all animals; these are the constant and necessary uses of the teeth and form the reason for their existence.

In many animals only one primary use is found, as in fishes and serpents, seizing the prey whole being all that is required; in the carnivora, seizing, dividing, and lacerating; in the herbivorous, seizing and triturating; while in man and the anthropoid apes all the primary uses are present.

While teeth for primary uses are universally found, many animals have teeth formed only for secondary uses. Examples of teeth used for attack and defense are found in the snout of the saw-fish, crowded with teeth, the incisor tusk of the mastodon, elephant, and male Narwhal, in the poison fang of the serpent—while the tusk of the male Narwhal characterizes its sex; as means of anchorage we have the two long projecting cusps of the mud-fish, which keep it stationary in the mud; as an aid to locomotion, the myxine, a parasitic fish, which uses its single, conical curved tooth to hold fast while its serrated tongue bores a channel for its passage into the tissue of the larger fish where it lives; for transporting and working of building materials we have the incisor teeth of the beaver; while for subserviency to speech man realizes the value of his teeth from the occasional loss of some anterior tooth.

Among the invertebrates, the annelids, insects and crustaceans use horny mandibles or jaws of various forms and in various ways, for seizing and dividing their food.

The leech, curiously, uses its three serrated jaws for scarifying any animal tissue and then sucks the blood.

The echinoderm possesses five sharp teeth placed in the wonderful apparatus called Aristotle's Lantern, which is used for breaking up the hard parts of the shell fish which constitute its food.

Of the mollusks, one author has said—" To describe the teeth of the molusks in detail would require several volumes."

In the conus and bela the teeth are arrow-shaped, sometimes barbed or accompanied by a poisonous secretion, forming a dangerous weapon. The fresh water snails have bent and deeply serrated teeth for tearing the vegetable matter used for food.

In the air-breathing mollusks some varieties approach the shape of vertebrate teeth and others are arranged with pavement-like regularity; these are used for masticating vegetable, and tearing animal matter used for food. The purpura drills a hole in the clam or oyster shell by a rotary motion of its radula and then sucks the fluids of the animal inside.

Among the fishes have been mentioned the parasitic myxine with its locomotive tooth and the mud-fish with its anchor teeth.

The sharp triangular teeth of the shark, in many rows, are used for seizing the small marine animals forming its food.

One variety, the cetacean, is very peculiar, possessing no cutting teeth, but only broad flat plates for crushing and triturating the shell-fish upon which it lives.

The wolf-fish has eight or nine pointed front teeth for tearing shell-fish from the rocks and three rows of blunt crushing teeth posteriorly for crushing their shells.

Among the osseous fish, those ordinarily met with, the prevailing type is the sharp pointed cuspid. The common pike is typical, possessing hundreds of sharp pointed teeth inclining backwards and inwards upon both jaws, including six or eight long, strong teeth for disabling and transfixing small fishes; all the struggles of the transfixed fish only alter its position favorably to the pike, when it is swallowed, usually head first, and often alive. A striking modification of this type is found in the angler, consisting in a row of large, hinged teeth on either jaw bending in, but not out.

